# The interplay between evolution, regulation and tissue specificity in the Human Hereditary Diseasome

**DOI:** 10.1186/1471-2164-11-S4-S23

**Published:** 2010-12-02

**Authors:** Shivashankar H Nagaraj, Aaron Ingham, Antonio Reverter

**Affiliations:** 1 CSIRO Livestock Industries, Queensland Bioscience Precinct, 306 Carmody Road, St. Lucia, Queensland 4067, Australia

## Abstract

**Background:**

Human disease genes can be distinguished from essential (embryonically lethal) and non-disease genes using gene attributes. Such attributes include gene age, tissue specificity of expression, regulatory capacity, sequence length, rate of sequence variation and capacity for interaction. The resulting information has been used to inform data mining approaches seeking to identify novel disease genes. Given the dynamic nature of this field and the rapid rise in relevant information, we have chosen to perform a single integrated mining approach to explore relationships among gene attributes and thereby characterise evolutionary trends associated with disease genes.

**Results:**

All against all cross comparison of 2,522 disease gene attributes revealed significant relationships existed between the age, disease-association and expression pattern of genes and the tissues within which they are expressed. We found that the over-representation of disease genes among old genes holds for tissue-specific genes, but the correlation between age and disease association vanished when conditioning on tissue-specificity. Of the 32 tissues studied, the genes expressed in pancreas are on average older than the genes expressed in any other tissue, while the testis expressed the lowest proportion of old genes. Following a focussed analysis on the impact of regulatory apparatus on evolution of disease genes, we show that regulators, comprising transcription factors and post-translation modified proteins, are over-represented among ancient disease genes. In addition, we show that the proportion of regulator genes is affected by gene age among disease genes and by tissue-specificity among non-disease genes. Finally, using 55,606 true positive gene interaction data, we find that old disease genes interacts with other old disease genes and interacting new genes interacts with genes originating from higher phylostrata.

**Conclusion:**

This study supports the non-random nature of the human diseasome. We have identified a variety of distinct features and correlations to other molecular attributes that can be used to distinguish the set of disease causing genes. This was achieved by harnessing the power of mining large scale datasets from OMIM and other databases. Ultimately such knowledge may contribute to the identification of novel human disease genes and an enhanced understanding of human biology.

## Background

Human diseases associated genes, collectively termed as the diseasome, are the focus of extensive studies. The consistent association of individual gene variants with disease has facilitated the decoding of the molecular basis of heritable diseases. On a larger scale, the diseasome itself has been scrutinized for characteristics that distinguish it from the remainder of the genome. Such studies on the diseasome have focussed on identifying causal mutations, mode of inheritance, their expression across a wide spectrum of tissues and to identify whether they are essential or non-essential genes (i.e. to test their embryonic lethality) [1]. For instance, it is well established that non-synonymous DNA mutations causing disease are atypical both in their rate and pattern of evolution [2], enriched at highly conserved amino acid positions [2] resulting in large changes in physicochemical properties of amino acids and seem likely to have severe effects on protein stability.

Following the availability of the human genome and an atlas of disease associated genes deposited in OMIM (Online Mendelian Inheritance in Man) [3] and other databases [4], researchers focussed on combining these datasets to gain insights into the evolution of the human diseasome. For instance, López-Bigas and co-workers [5,6] classified over 1,600 human disease genes from various databases and studied their properties using Gene Ontology (GO) terms and expression across different tissues. They observed that the functional pattern of genes causing dominant and recessive diseases is markedly different and report extensive correlations between disease gene attributes. Based on these findings, they proposed that the division of human diseasome by mode of inheritance (dominant or recessive) could enhance both understanding of the disease process and prediction of candidate disease genes.

Furthermore, a number of groups applied comparative genomic approaches to study evolution of disease genes. For instance, Huang *et al*[7] investigated whether human disease genes differ significantly from their rodent orthologs with respect to their overall levels of conservation and their rates of evolutionary change. Following comparison of human genome with mouse and rat genome sequences for disease genes, they report that most (99.5%) human disease genes have been retained in rodent genomes. Using a large set of disease and non-disease genes, Smith *et al*[2] compared human with rodents to infer evolutionary patterns. They measured Ka, Ks and Ka/Ks between disease and non-disease genes in human and found significant difference in Ka/Ks ratios. They also describe the evolution of disease and non-disease genes based on protein structure, gene length and tissue specificity. Applying various *in silico* approaches, Kondrashov et al [8] compared 18 crucial parameters among 1,273 human disease genes and reported that disease genes evolve more slowly, have wider phylogenetic distributions, code for longer proteins containing more alanine and glycine and less histidine, lysine and methionine, possess larger numbers of longer introns with more accurate splicing signals and have higher and broader expressions.

Phylostratigraphic (ps) approach is a method to quantify and statistically analyze gene emergence at different levels of the taxonomic hierarchy. The approach is based on the assumption that at least a significant fraction of genes has retained their function after origination [9]. This approach was developed by Domazet-Lošo et al [9] to uncover the genomic history of major adaptations in metazoans. Later, the authors used this ps approach [10] to study the evolution of human genes and reported that disease genes tend to be ‘old’ rather than ‘young’. In this study, authors also suggested that genetic disease is an inescapable component of evolution. This is somewhat counterintuitive because disease-associated genes would be expected to be selected against by natural evolution and rapidly eliminated from the population and not maintained over extended periods of time. Alternatively, disease-associated genes may have previously evolved to participate in highly-regulated processes that resulted in optimal conditions for a given environment, but currently these functions are no longer seen as optimal anymore but detrimental. Independently, we have investigated the impact of tissue specificity and differential gene network connectivity on disease associations of human genes [11]. We found that disease associated genes are more likely to show tissue specific expression and most frequently interact with other disease genes. Based on these biological properties, we developed a guilt-by-association algorithm that lead to the discovery of a group of 112 non-disease annotated genes that predominantly interact with disease-associated genes, impacting on disease outcomes [11].

In the present study, and in order to further characterise evolutionary trends associated with disease genes, we have integrated data generated in previous studies enabling us to compare a greater number of attributes, generated using current technologies (massively parallel signature sequencing (MPSS) data generated from 32 tissues) combined with the latest version of OMIM. Specific attributes tested for relationships include gene age, disease, tissue specificity, known interactions, sequence length, chromosome location and whether or not a gene product acts as a regulator by either being a transcription factor (TF) and/or harbouring post-translational modification (PTM) sites.

## Results and discussion

In order to identify novel, and biologically meaningful, interconnections among disease genes, we carried out all against all cross comparison of 2,522 disease genes with a number of relevant molecular attributes (see Methods) and raised several queries within the framework of evolution of disease genes. Table 1 shows the breakdown of 12,753 genes within each category with respect to age of the genes, tissue specificity, gene-gene interactions, gene regulation, and association with disease. Significant findings from each of these comparisons are summarised in Table 2 (and Additional file 1) and discussed in the following sections. As this study aims at all against all relationships, the individual gene attributes and its relationship with other attributes are described simultaneously. For instance, while discussing the impact of tissue specificity on disease genes, we also describe the impact of regulators and/or gene length that affect tissue specificity.

**Table 1:** Summary of the overall gene based datasets analysed during this analysis. The numbers shown in the table represents the genes in respective categories.

	1. Age		2. Specificity		3. Expression		4. Interactions		5. Disease		6. Tr. Factor		7. PTM		9. Sequence	
	OLD	NEW	TSP	HKP	EXL	EXH	INT	NIN	DIS	NDI	TFA	NTF	PTM	NPT	SHO	LON
OLD	8301		2424	2648	2587	2485	3445	4856	1892	6409	650	7651	1310	6991	3362	4939
NEW		4452	1204	700	1012	892	1213	3239	630	3822	349	4103	475	3977	3020	1432
TSP			3628		2619	1009	1571	2057	879	2749	304	3324	582	3046	1719	1909
HKP				3348	980	2368	1783	1565	848	2500	347	3001	737	2611	1144	2204
EXL					3599		1543	2056	830	2769	342	3257	565	3034	1512	2087
EXH						3377	1811	1566	897	2480	309	3068	754	2623	1351	2026
INT							4658		1524	3134	573	4085	1335	3323	1963	2695
NIN								8095	998	7097	426	7669	450	7645	4419	3676
DIS									2522		262	2260	607	1915	988	1534
NDI										10231	737	9494	1178	9053	5394	4837
TFA											999		228	771	493	506
NTF												11754	1557	10197	5889	5865
PTM													1785		668	1117
NPT														10968	5714	5254
REG															1090	1466
NRE															5292	4905
SHO															6382	
LON																6371

Abbreviations: OLD: Old gene (ps1 and ps2); NEW: New genes (ps3 to ps19); TSP: Tissue specific genes; HKP: House Keeping genes; EXL: Expression Low; Expression High; INT: Interacting genes; NIN: Non-interacting genes; DIS: Disease-associated genes; NDIS: Non disease associated genes; TFA: Transcription Factors; NTF: Non-transcription Factors; PTM: Genes with at least one post-translational modification; NPT: Genes with no post-translational modification; LONG: Genes longer than 24kb in length; REG: Genes with regulatory role TF and/or PTM; NRE: Non-regulatory genes. SHO: Genes shorter than 24kb in length.

**Table 2:** Summary of the significant relationships between the gene attributes.

Disease	Age	PTMs	Tissue Specificity	Interactions
When compared to non-disease genes a higher proportion of Disease genes are Interacting (20) • PTM (13) • Long (13) • Old (8)	When compared to New genes a higher proportion of Old genes are • Long (27) • House Keeping genes (16) • Interacting (14) • PTM (6)	When compared to proteins that carry no PTM a higher proportion of Protein that harbour PTMs are • Interacting (23) • Long (15) • Expressed at high levels (7) • House keeping genes (6)	When compared to proteins that carry no PTM a higher proportion of Protein that harbour PTMs are • Interacting (23) • Long (15) • Expressed at high levels (7) • House keeping genes (6)	When compared to non-interacting genes a higher proportion of Interacting are • Long (12) • Expressed at high levels (10) • Transcription factors (7)

### 1. The interplay between evolution, disease genes and tissue specificity

In a bid to elucidate the complex association among disease and non-disease genes, old and new genes and housekeeping and tissue-specific genes, we integrated functional gene attribute information spanning 12,753 genes. OLD and NEW genes represented 66 and 34% of all genes, respectively. DIS and NDIS genes represented 24% and 76%, respectively. We observed that house-keeping genes are over-represented among old genes; that tissue specific genes are over-represented among disease genes; and that while disease genes are over-represented among old genes, this over-representation is more apparent among tissue specific genes. Overall, we find that the age attribute has a strong correlation with all the other gene attributes used in this study such as tissue-specificity, gene-gene interactions, gene length and regulatory nature of genes.

#### a. The impact of old genes and new genes over tissue-specificity

Old genes are ubiquitously expressed across tissues, more associated with diseases and are more likely to be regulators (Additional file 2: Table S2). There is in fact a 1.32-fold higher occurrence of a regulatory function among old genes (P < 0.0001). This decrease in regulatory function in new genes is counterintuitive as it occurs when there is a significant increase in complexity of organisms. This result is however consistent with the idea that there is an upper limit to TF-based regulation and warrants the emergence of novel regulatory mechanisms. It is now clear that in higher eukaryotes non-coding RNAs function as this alternative mechanism [12]. However, tissue-specificity, as measured by the proportion of tissues in which a gene is expressed out of a total of 32 tissues represented, drives many of the above-mentioned relationships. In essence, conditioning on tissue-specificity nullifies the correlations between gene age and the other variables, except for the correlation between age and sequence length, meaning older genes are still longer (Additional file 2: Table S3).

#### b. Disease genes and tissue-specificity

Disease genes are over-represented among old, interacting and genes involved in regulation and this over-representation is more pronounced among tissue-specific genes. For instance, 31.2% disease genes are involved in regulation compared to only 16.9% disease genes among the non-regulatory genes. In addition, the strong correlation between disease genes and regulatory genes is intact even when conditioning on tissue-specificity. The correlation between tissue specificity and disease-association has also been reported by other groups [13,14]. Specifically, using a 2,400 human-rodent orthologs and 834 rat-mouse orthologs and EST information, Duret and Mouchiroud [15] observed that tissue-specific genes exhibited higher K_A_/K_S_ ratios than housekeeping genes. Confirming this finding, Huang et al [7] demonstrated that much of this effect is explicable in terms of a correlation between a gene's tissue-specificity and the cellular localization of its encoded protein. In this study, we found a 1.54 over-representation of disease genes among tissue specific old genes compared to new genes. This relationship was not impacted by tissue-specificity in new genes.

### 2. The evolution of the regulatory diseasome

Post-transcriptional regulation by transcription factors (TF) at the gene level [16] and post-translational modifications (PTM) [17] at the protein level are two principal regulatory mechanisms in eukaryotes. One of the key components of this study was the consideration of regulatory function in the context of disease and evolution. Specifically, we evaluated the role of regulators during the course of evolution of disease genes by utilizing TF and PTM data. Firstly, we analysed regulators by combining TF and PTM to explore disease genes from a regulatory viewpoint. Next, we separated the TF and PTM data to assess their independent effect during the course of evolution represented by respective phylostrata.

We found that genes encoding proteins with PTMs are over-represented among old genes, housekeeping genes, and interacting genes and in TF themselves. In addition, of the 228 genes that have both PTMs and are TF, 72 are disease genes (32% against 20% in all genes). Out of these 228 PTMs and TFs, 30 (13%) originated in ps5 (metazoa) compared to only 2% of total genes formed in ps3 demonstrating significant inclusion of regulatory modules to metazoans. This result is not surprising as metazoans by definition are multicellular organisms and would undoubtedly require additional levels of regulation to ensure accurate morphological development. Finally, the proportion of regulators is affected by age among disease genes (with ~ 1/3 more regulator genes among old genes compared to new genes), while it is affected by tissue-specificity among non-disease genes (also with ~1/3 more regulators among housekeeping genes compared to tissue-specific genes) (Additional file 2: Table S4).

**Figure 1:**
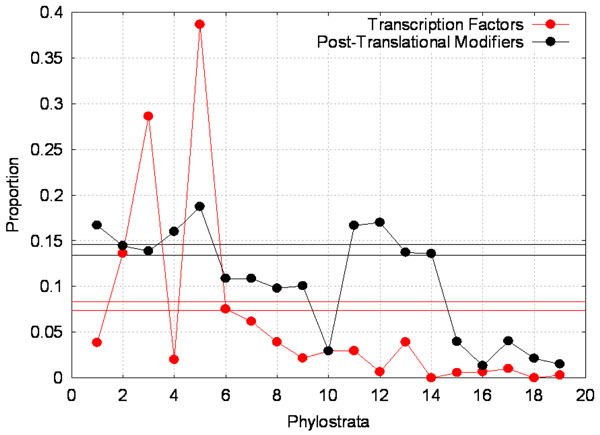
**Distribution of transcription factors and proteins that undergo post-translational modification across the phylostrata.** Proportion of transcription factors (TF; red profile) and post-translational modifiers (PTM; black profile) originating at each of the 19 phylostrata. Horizontal red and black lines correspond to the bounds of the 95% confidence interval for TF and PTM, respectively, and based on the entire set of 12,753 genes.

When regulatory apparatus was studied separately for TF and PTM (see Figure 1), we found an over-representation of TF originating in ps3 (fungi) at 28.6% compared to only 7.8% of all genes being TF. We did not observe an over-representation of PTM originating at ps3. This suggests that the evolution from eukaryotes to fungi requires a substantial increase in transcriptional regulation, while post-translational modification mechanisms are less relevant. In contrast, at ps5 (metazoa), a significant over-representation of both TF and PTM was clearly apparent. PTMs experienced another surge at ps11 (vertebrata) that was not coupled with a surge in TF, indicating significant increase in protein level regulation post vertebrates during the course of evolution.

Other less understood eukaryotic regulatory mechanisms such as CpG island methylation was not included in the analysis. Although CpG island methylation plays an important role in epigenetic gene control in general via hyper and hypo methylation of regulatory regions (promoters) their precise role across the ‘diseasome’ is not well established. For instance, there are independent studies focussed on cancer [18] or methylation patterns within the Major Histo-compatibility Complex (MHC) as a part of human epigenome project (http://www.epigenome.org/).

### 3. The pattern of gene-gene interactions among gene attributes

We looked at the combined effect of age (old vs. new), disease-association (yes vs. no) tissue-specificity and regulatory condition (yes vs. no) on the number of interactions. By and large, age had the highest effect with Old genes having ~50% more connections than New genes (9.227 vs. 6.1245; P < 0.0001). Regulatory role had the second highest effect, with regulators having ~40% more connections than non-regulators (8.980 vs. 6.371; P < 0.0001). Importantly, after adjusting for these two variables (age and regulatory conditions), neither tissue-specificity nor disease-association were found to be significant (P > 0.05) sources of variation in the number of connections observed for a given gene. However, there was a tendency for DIS genes to have more connections than NDIS genes (8.145 vs. 7.206; P = 0.0675), and for HK genes to have more connections than TS genes (8.109 vs. 7.242; P = 0.0855). While the original study of Domazet-Loso and Tautz [10] failed to find a significant re-ranking of gene functional ontologies when comparing disease and non-disease genes in each of the phylostrata, the incorporation of 55,606 true positive interactions among 7,197 genes [19] allowed us to further explore these relationships. Three major findings were of particular relevance: Firstly, there was a bias in the way genes interact, with interacting old genes more likely to interact with other old genes; Secondly, interacting new genes are more likely to interact with other new genes; Finally, we observed that new genes interact more cohesively than old genes.

### 4. The non-random distribution of genes along the genome

**Figure 2:**
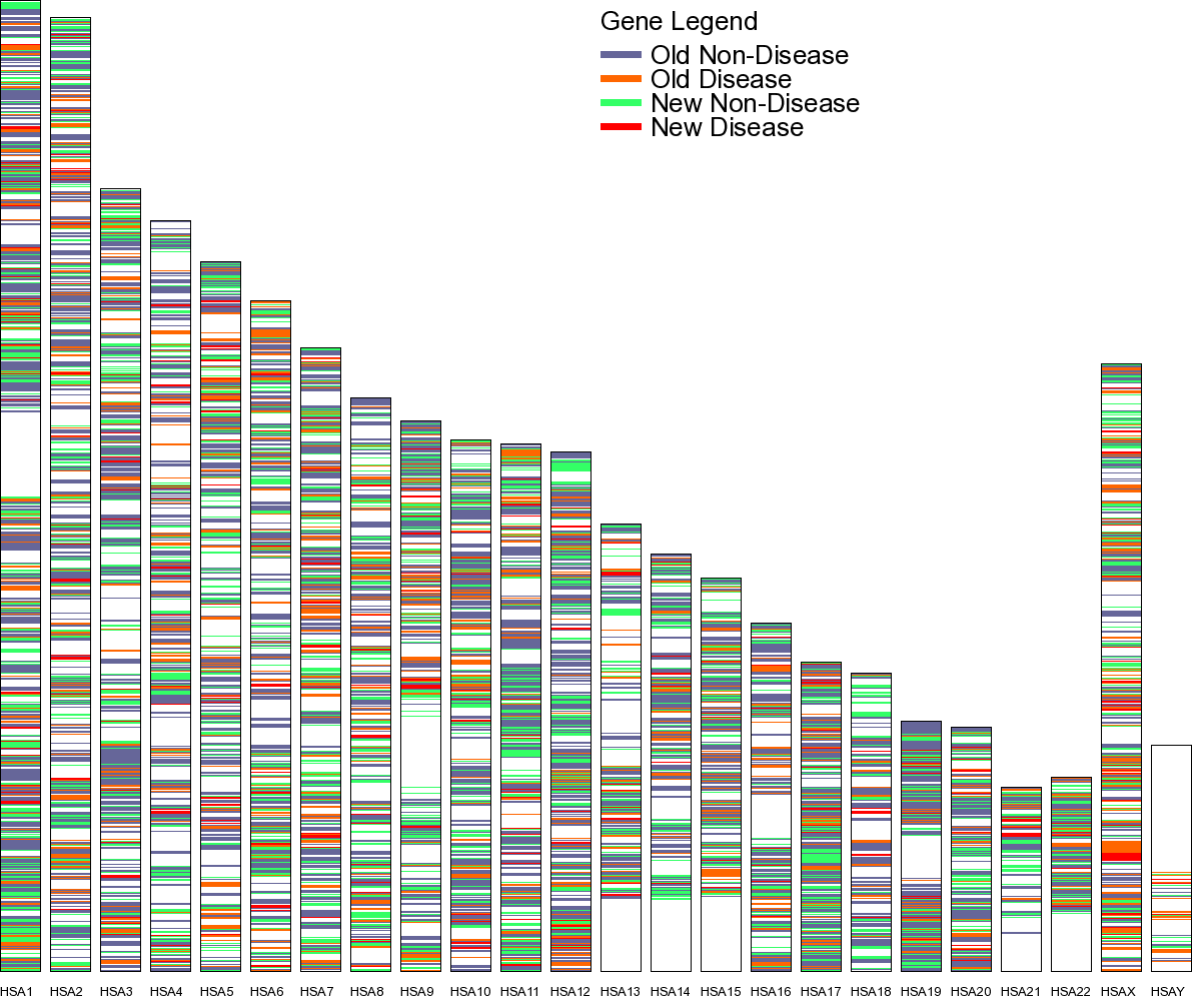
**Chromosomal distribution of genes based on their evolutionary age and disease status**. Each colour represent a class; Old-Non-Disease, Old-Diseased, New-Non-Disease and New-Diseased. Choromosome 19 comprises of least number of disease genes where as choromosome X contains highest number of disease genes.

**Figure 3:**
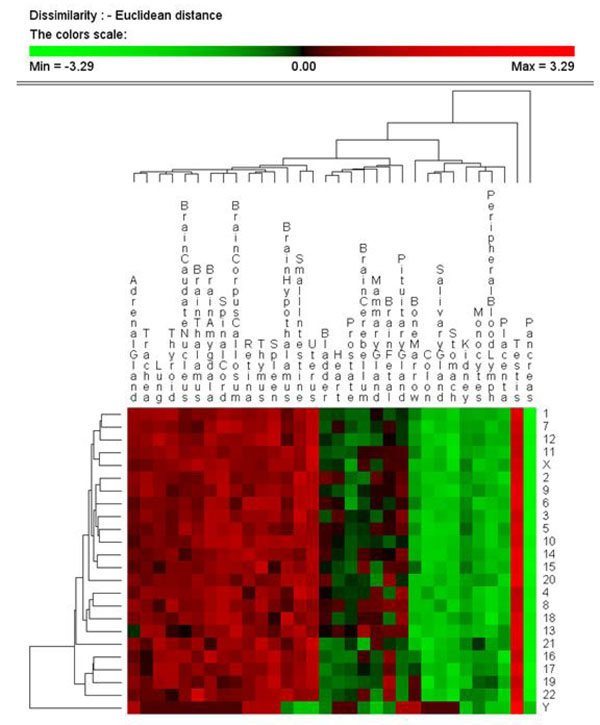
**Hierarchical clustering of gene expression in 32 tissues with all human chromosomes.** For each chromosome the tissues in which each of its genes is expressed was recorded. The picture shows the hierarchical clustering of chromosomes (rows) and tissues (columns) based on the number of genes located in each chromosome and expressed in each tissue. Spectrum goes from green to red for low and high number of genes. The figure reveals an anatomically sensible cluster of tissues.

Genes are non-uniformly distributed in the human genome and we asked whether this heterogeneity may further our understanding of evolutionary trends among disease genes. Consequently, we produced a chromosomal distribution of genes according to their evolutionary age and disease status (Figure 2). The chromosomal locations of all the genes were mapped to the genome according to their evolutionary age and disease status and classified into four categories: Old-Non-Disease, Old-Disease, New-Non-Disease and New-Disease. We noted that the sex chromosomes have a higher proportion of disease genes; 28.7 ± 1.9 % of genes in X and 38.3 ± 8.4 % of genes in Y, compared to only 13.7 ± 1.1 % of genes in Chromosome 19. This disproportionately high number of hereditary diseases for the X chromosome was well documented during analysis of its genome sequence [20]. In contrast, Chromosome 19 has the lowest proportion of disease genes, the shortest genes (average length 24.1 ± 1.3 kb) and tissue-specificity comparable to the X and Y sex chromosomes. This was attributed to Chromosome 19 having the highest gene density of all chromosomes, more than double the genome-wide average [21]. Expanding this analysis, we tested whether there was a chromosome whose genes have a higher than average specificity for a given tissue (see Figure 3). This analysis revealed that all chromosomes seem to have a large proportion of genes expressed in testis and a low proportion of genes expressed in pancreas. Additionally, for some tissues there was a disproportionate representation of genes from a single chromosome. As an example, we found an over-representation of genes expressed in cerebellum located on chromosome 13. From this we predict that chromosome 13 may be a potential target to study central nervous system (CNS) diseases. A search of the literature confirms that deletions in chromosome 13 impacting cerebellum development have been identified (see McCormack et al. [22] and references therein) and as such further justifies fine mapping approaches in the search for QTL.

### 5. Expression patterns and gene attribute distribution across tissues

**Figure 4:**
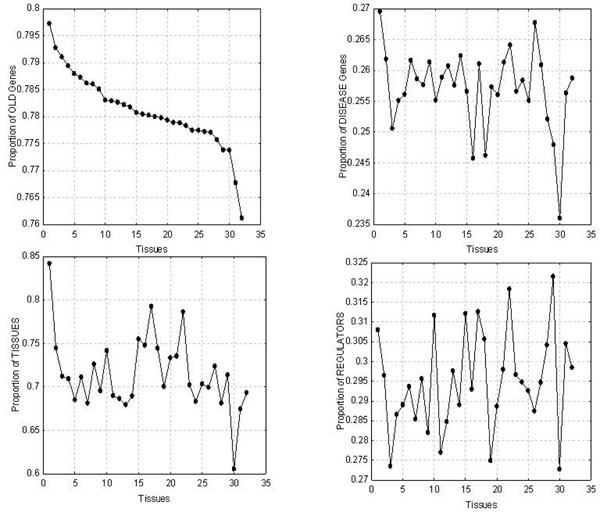
**The evolution of tissues based on gene expression across different tissues** . Genes expressed in each of the 32 tissues are recorded and plotted are the proportion of old genes (upper left panel; old defined as phylostrata 1 or 2), proportion of disease genes (upper right panel), proportion of other tissues where the genes are also expressed (bottom left panel) and the proportion of regulator genes (bottom right panel). Their identity and percentage of genes they expressed (out of 7,111) are as follows: 1. Pancreas (23.7%); 2. Kidney (41.6%); 3. Pituitary gland (49.8%); 4. Cerebellum (48.1%); 5. Fetal brain (50.3%); 6. Adrenal gland (50.4%); 7. Amygdala (56.4%); 8. Heart (48.0%); 9. Brain caudate nucleus (53.8%); 10. Peripheral blood lymphocytes (38.1%); 11. Thalamus (54.4%); 12. Spinal cord (57.0%); 13. Uterus (58.8%); 14. Brain corpus callosum (55.7%); 15. Placenta (37.5%); 16. Colon (35.3%); 17. Stomach (34.8%); 18. Monocytes (40.4%); 19. Hypothalamus (54.3%); 20. Prostate (46.0%); 21. Bladder (48.3%); 22. Salivary gland (36.9%); 23. Thyroid (54.8%); 24. Small intestine (57.3%); 25. Trachea (54.0%); 26. Retina (55.0%); 27. Mammary gland (48.5%); 28. Thymus (56.6%); 29. Bone marrow (41.9%); 30. Testis (65.1%); 31. Spleen (56.9%); 32. Lung (55.1%).

By associating gene expression patterns across 32 tissues to molecular attributes used in this study (old, new, disease, non-disease, etc.) we were able to provide novel insights into possible trends in evolution of various tissues from a diseasome viewpoint. In other words, any non-uniform proportions of disease and non-disease genes may unravel the complexity of evolution of tissues and their association with diseases (pancreatic cancer, Alzheimer’s, etc). Therefore, for each of the 32 tissues, we explored the proportion of (1) old genes (ie. those that evolved in ps1 or ps2); (2) disease genes; (3) number of tissues where the genes are expressed; and (4) regulators in each tissue (Figure 4). We found that the genes expressed in pancreas are on average older than the genes expressed in any other tissue, have a higher association with disease and are expressed in more tissues, suggesting that the pancreas could be one of the first discrete tissues to evolve. This finding is in agreement with a phylogeny and ontogeny based study of the pancreas wherein authors propose that the pancreas is indeed one of the first tissues to evolve [23]. Conversely, genes expressed in the lungs are on average the newest, but this is not the tissue with the lowest proportion of disease genes. Instead, the lowest proportion of disease genes was found in the testis followed by the trachea and bone marrow. This finding is supported by another study reporting the majority of retrogenes (new genes formed through gene duplication) are specifically expressed in testis, whereas their parental genes show broad expression patterns [24]. It should be noted with caution that expression of disease genes cannot be used as a parameter to asses whether these tissues are prone for diseases. In other words, this data is insufficient to conclude that the pancreas is more prone to disease than the testis. Further, the testis was also found to have the highest proportion of tissue-specific genes. Finally, the retina showed a high proportion of disease genes (second only to pancreas) even though it ranks among the newest tissues.

## Conclusion

We have used a systems biology approach to integrate large relevant datasets to yield novel, meaningful biological insights into the evolution and tissue-specificity of disease genes. Through the incorporation of tissue-specificity attribute, we found that the recently documented over-representation of disease genes among old genes is certainly true for tissue-specific genes, while among housekeeping genes this relationship vanishes. Nevertheless, we acknowledge the challenge of establishing error-free relationships due to the myriad of possible interactions that could exist among large numbers of heterogeneous variables.

Researchers have intuitively focused on aberrations in regulatory genes as a likely basis for the disease development. Our findings support this approach because disease genes were found to be over-represented in both the PTM and TF regulatory categories. Finally, this study represents a conceptual scaffold for dissecting human diseasome and reveals novel correlations among molecular attributes, some known, but many unexpected, that might be helpful in the identification of novel genes disrupted in diseases.

## Methods

When discussing gene attributes, a notation consistent with Domazet-Loso and Tautz [10] is used where old refers to old genes, originating in ps1 or ps2 (i.e., up to eukaryotes and before fungi); new genes (ps3 to ps19); Tissue-specific genes (expressed in < 14 tissues); House-keeping genes (expressed in ≥ 14 tissues); Short/Long: Genes shorter/longer than 24 kb in length; Regulatory genes: Genes with regulatory role, they are either transcription factors or and/or harbour at least one post-translational modification in encoded protein.

## Data integration and assembly

Our study uses the following distinct data sets:

1. The list of human genes along with their phylostrata of origin was downloaded from (Domazet-Loso and Tautz [10]) (Ensembl version 45 -22,937 unique proteins).

2. Expression data from massively parallel signature sequencing (MPSS) covering 182,719 tag signatures across 32 tissues [25].

3. These two datasets were merged to form a single list of 12,753 genes with phylostrata of origin and expression abundance.

4. The complete list of TFs was retrieved from BiblioSphere [26]. Data for PTM for human data were obtained from the most recent version of Human Protein Reference Database (HPRD – Release 7) [27]. Although TF and PTM bring about regulatory mechanisms at different phases of cellular process, we have combined TF and PTM and refer to them as simply regulators in order to capture the maximum regulatory apparatus in eukaryotes.

5. The interaction data comprising 55,606 true positive interactions among 7,197 genes were downloaded from functional studies [19].

6. The information for disease association of genes was obtained from OMIM (Online Mendelian Inheritance in Man) database [3] with 2,522 of them defined as disease-causing (i.e., associated with either known disease phenotype or polymorphic sequence known).

AWK and Perl scripts were written to assemble and analyse data on a Linux server.

## Statistical analyses and significance

When relationships were based on Pearson correlation and partial correlation coefficients, statistical significance was assessed using the Procedure CORR of SAS version 9.1.3 (SAS Institute Inc., Cary, NC, USA).

When proportions were being compared, a two-tailed z-test for the difference between two proportions was performed assuming unequal group variances as described by AP Statistics Tutorial (http://stattrek.com/AP-Statistics-4/Test-Difference-Proportion.aspx). As a guide, the reader is reminded that having any two proportions each computed with more than 1,000 records, if these two proportions differ by more than 2% then the difference is significant at P < 5% significance (or 95% confidence).

Least square means for the number of connections across the levels of the various class variables were obtained from fitting linear models using the Procedure GLM (General Linear Models) of SAS version 9.1.3 (SAS Institute Inc., Cary, NC, USA). A two-sided t-test was used to test the significance of the difference between two least square means of interest (eg. connections in DIS vs connection in NDIS genes).

## Abbreviations

MPSS: massively parallel signature sequencing; OMIM: Online Mendelian Inheritance in Man

## Competing interests

The authors declare that they have no competing interests.

## Authors' contributions

AR conceived the study, SHN and AR carried out the data mining approaches and drafted the manuscript. AI directed the design and coordination of the biological relevance of the results and drafted the manuscript. All authors read and approved the final manuscript.

## Supplementary Material

Additional file 1 Table S1. Summary of the significant relationships between the gene attributes: Contingency table for 12,753 Genes. Click here for file

Additional file 2 Gene distribution into phylostrata and related statistical data.
Table S2. Distribution of the 12,753 genes into different phylostrata (PS) represented in percentages
Table S3. Pearson Partial Correlation Coefficients conditional on tissue specificity of genes
Table S4. Mean and standard error computations for gene length, regulators and interactors Click here for file
