# Supplementary material for: The interplay between evolution, regulation and tissue specificity in the Human Hereditary Diseasome
Source: BMC Genomics. 2010 Dec 2;11(Suppl 4):S23. doi: 10.1186/1471-2164-11-S4-S23 (PMC3005915; doi:10.1186/1471-2164-11-S4-S23)
Supplement: Additional file 1 — Table S1. Summary of the significant relationships between the gene attributes: Contingency table for 12,753 Genes. [file 1471-2164-11-S4-S23-S1.pdf]

The interplay between evolution, regulation and tissue specificity in the Human Hereditary Diseasome

Shivashankar H Nagaraj , Aaron Ingham and Antonio Reverter

Additional file 1

Table S1. Summary of the significant relationships between the gene attributes: Contingency table for 12,753 Genes.

	1. Age		2. Specificity		3. Expression		4. Interactions		5. Disease		6. Tr. Factor		7. PT. Modif.		8. Sequence	
	OLD	NEW	TSP	HKP	EXL	EXH	INT	NIN	DIS	NDI	TFA	NTF	PTM	NPT	SHO	LON
OLD			2424<br>47	2648<br>53	2587<br>51	2485<br>49	3445<br>41	4856<br>59	1892<br>22	6409<br>78	650<br>8	7651<br>92	1310<br>16	6991<br>84	3362<br>40	4939<br>60
NEW			1204<br>63	700<br>37	1012<br>53	892<br>47	1213<br>27	3239<br>73	630<br>14	3822<br>86	349<br>8	4103<br>92	475<br>10	3977<br>90	3020<br>67	1432<br>33
TSP					2619<br>72	1009<br>28	1571<br>43	2057<br>57	879<br>24	2749<br>76	304<br>8	3324<br>92	582<br>16	3046<br>84	1719<br>47	1909<br>53
HKP					980<br>29	2368<br>71	1783<br>53	1565<br>47	848<br>25	2500<br>75	347<br>10	3001<br>90	737<br>22	2611<br>78	1144<br>34	2204<br>66
EXL							1543<br>43	2056<br>57	830<br>23	2769<br>77	342<br>9	3257<br>91	565<br>15	3034<br>85	1512<br>42	2087<br>58
EXH							1811<br>53	1566<br>47	897<br>26	2480<br>74	309<br>9	3068<br>91	754<br>22	2623<br>78	1351<br>40	2026<br>60
INT									1524<br>32	3134<br>68	573<br>12	4085<br>88	1335<br>28	3323<br>72	1963<br>42	2695<br>58
NIN									998<br>12	7097<br>88	426<br>5	7669<br>95	450<br>5	7645<br>95	4419<br>54	3676<br>46

DIS											262<br>10	2260<br>90	607<br>24	1915<br>76	988<br>39	1534<br>61
NDI											737<br>7	9494<br>93	1178<br>11	9053<br>89	5394<br>52	4837<br>48
TFA															493<br>49	506<br>51
NTF															5889<br>50	5865<br>50
PTM															668<br>37	1117<br>63
NPT															5714<br>52	5254<br>48

OLD = ps <3; TSP <14 tissues; EXL <30 tpm; REG = TF and/or PTM; SHO < 24 Kb

Abbreviations: OLD: Old gene (ps1 and ps2); NEW: New genes (ps3 to ps19); TSP: Tissue specific genes; HKP: House Keeping genes; EXL: Expression Low; Expression High; INT: Interacting genes; NIN: Non-interacting genes; DIS: Disease-associated genes; NDIS: Non disease-associated genes; TFA: Transcription Factors; NTF: Non-transcription Factors; PTM: Genes with at least one post-translational modification; NPT: Genes with no post-translational modification; LONG: Genes longer than 24kb in length; REG: Genes with regulatory role TF and/or PTM; NRE: Non-regulatory genes. SHO: Genes shorter than 24kb in length.
