# Supplementary material for: The interplay between evolution, regulation and tissue specificity in the Human Hereditary Diseasome
Source: BMC Genomics. 2010 Dec 2;11(Suppl 4):S23. doi: 10.1186/1471-2164-11-S4-S23 (PMC3005915; doi:10.1186/1471-2164-11-S4-S23)
Supplement: Additional file 2 — Gene distribution into phylostrata and related statistical data. Table S2. Distribution of the 12,753 genes into different phylostrata (PS) represented in percentages Table S3. Pearson Partial Correlation Coefficients conditional on tissue specificity of genes Table S4. Mean and standard error computations for gene length, regulators and interactors [file 1471-2164-11-S4-S23-S2.pdf]

The interplay between evolution, regulation and tissue specificity in the Human Hereditary Diseasome

Shivashankar H Nagaraj , Aaron Ingham and Antonio Reverter

Additional file 2: Gene distribution into phylostrata and related statistical data.

Table S2. Distribution of the 12,753 genes into different phylostrata (PS) represented in percentages

PS	Genes(No)	Genes	Tissues	EXP	INT	DIS	TF	PTM	REG	Length(kb)
1	4924	38.611	46.982	1.553	45.268	26.381	3.859	16.694	19.496	75.871
2	3377	26.480	51.989	1.525	36.008	17.560	13.622	14.451	25.407	68.984
3	339	2.658	49.743	1.526	32.153	21.534	28.614	13.864	37.168	54.706
4	200	1.568	38.750	1.476	38.500	22.000	2.000	16.000	17.500	106.135
5	251	1.968	41.146	1.504	54.980	27.888	38.645	18.725	45.418	67.806
6	1315	10.311	37.615	1.514	31.483	14.297	7.529	10.874	16.426	37.832
7	469	3.678	40.692	1.559	26.439	18.124	6.183	10.874	15.565	41.825
8	51	0.400	34.615	1.508	15.686	9.804	3.922	9.804	11.765	37.704
9	139	1.090	38.965	1.492	28.777	11.511	2.158	10.072	12.230	48.950
10	34	0.267	43.750	1.729	41.176	14.706	2.941	2.941	5.882	26.134
11	270	2.117	32.468	1.578	27.778	15.556	2.963	16.667	18.889	32.328
12	300	2.352	32.106	1.547	41.000	14.333	0.667	17.000	17.667	24.004
13	51	0.400	40.625	1.727	15.686	15.686	3.922	13.726	17.647	14.002
14	81	0.635	32.340	1.477	28.395	8.642	0.000	13.580	13.580	28.748
15	176	1.380	21.610	1.630	11.364	8.523	0.568	3.977	4.545	13.296
16	299	2.345	25.824	1.542	9.030	4.013	0.669	1.338	2.007	12.302
17	99	0.776	22.604	1.507	7.071	4.040	1.010	4.040	4.040	11.679
18	47	0.369	27.455	1.441	2.128	8.511	0.000	2.128	2.128	18.927
19	331	2.595	20.184	1.355	1.511	2.719	0.302	1.511	1.813	13.026

Abbreviations: PS: phylostrata; EXP: Expression; INT: Interaction; DIS: Disease-associated genes; TF: Transcription Factor; PTM: Post-translational modification; REG: Genes with regulatory role TF and/or PTM.

Table S3: Pearson Partial Correlation Coefficients (Conditional on Tissue Spec), N = 19 (correlations with an asterisk indicate different from zero, P < 0.01)

	PS	DIS	TF	PTM	REG	Length of the gene
**PS**	1.00000	-0.54056	-0.20208	-0.42417	-0.35111	-0.74956*
**DIS**	-0.54056	1.00000	0.42506	0.62636*	0.63416*	0.55893
**TF**	-0.20208	0.42506	1.00000	0.19070	0.84955*	0.11071
**PTM**	-0.42417	0.62636	0.19070	1.00000	0.67386*	0.40810
**REG**	-0.35111	0.63416*	0.84955*	0.67386*	1.00000	0.27558
**Length of the gene**	-0.74956*	0.55893	0.11071	0.40810	0.27558	1.00000

Table S4: Mean and standard error computations for gene length, regulators and interactors

Category	Number of genes	Length		Regulators		Interactors	
		Mean	Standard Error	Mean	Standard Error	Mean	Standard Error
OLD__TS__NDI	1767	79.394	3.039	19.864	0.949	38.653	1.159
OLD__TS__DIS	657	92.386	6.710	29.376	1.778	65.906	1.851
OLD__HK__NDI	1956	80.325	2.696	27.505	1.010	49.693	1.131
OLD__HK__DIS	692	95.379	4.842	31.936	1.774	69.075	1.758
NEW__TS__NDI	982	41.405	3.518	18.534	1.241	31.365	1.481
NEW__TS__DIS	222	61.285	9.508	42.342	3.324	66.216	3.182
NEW__HK__NDI	544	51.114	3.281	26.287	1.889	42.279	2.120
NEW__HK__DIS	156	78.797	9.881	41.667	3.960	66.026	3.804

Abbreviations: OLD__TS__NDI : Old , tissue specific, non-disease; OLD__TS__DIS: Old , tissue specific, disease; OLD__HK__NDIS: Old, house-keeping, non-disease; OLD__HK__DIS: Old, house-keeping, disease; NEW__TS__NDIS: New, tissue specific, non-disease; NEW__TS__DIS: New tissue specific, disease; NEW__HK__NDIS: New, house-keeping, non-disease; NEW__HK__DIS: New, house-keeping, disease
